# Griscelli Syndrome Type 2 Revealed by Macrophage Activation Syndrome: Two Cases From the Same Family

**DOI:** 10.7759/cureus.93180

**Published:** 2025-09-25

**Authors:** Boutayna Halimy, Abdelilah Radi, Azzeddine Laaraje, Hassani Amale, Rachid Abilkassem

**Affiliations:** 1 Pediatric Department, Mohammed V Military Training Hospital, Mohammed V University, Rabat, MAR

**Keywords:** griscelli syndrome, hereditary disease, immune deficiency, macrophage activation syndrome, partial albinism

## Abstract

Griscelli syndrome type 2 is a rare autosomal recessive disorder caused by mutations in the RAB27A gene. It is characterized by partial albinism, a silvery sheen of the hair, and an immune deficiency.

We report cases of two siblings, a seven-year-old boy and his 10-year-old sister, who were admitted to the emergency department with sepsis complicated by macrophage activation syndrome. Their clinical course was rapidly unfavorable.

The diagnosis of Griscelli syndrome type 2 was made in the light of a combination of clinical and biological arguments: oculocutaneous hypopigmentation, silvery sheen of the hair, the absence of psychomotor delay, the occurrence of a macrophage activation syndrome following an infection, and especially the pathognomonic appearance on microscopic examination of a hair sample. The absence of giant granulations in the nucleated cells made it possible to eliminate Chediak-Higashi syndrome.

Griscelli syndrome type 2 should be considered in children presenting with hypopigmentation, silvery hair, and immune dysregulation, particularly when complicated by macrophage activation syndrome.

## Introduction

Griscelli syndrome (GS) is a rare, autosomal recessive disorder first described by Griscelli et al. in 1978, with approximately 160 cases reported worldwide [1]. It is divided into three types, each caused by mutations in distinct genes, i.e., MYO5A (type 1), RAB27 (type 2), and MLPH (type 3), which determine the clinical manifestations.

A characteristic feature of all GS types is partial albinism, with a silvery hair and eyebrow appearance. Type 1 is primarily associated with severe neurological impairment, type 3 presents as an isolated pigmentary disorder, and type 2 is notable for profound immune dysfunction, which may precipitate macrophage activation syndrome (MAS), a severe hyperinflammatory reaction characterized by uncontrolled activation of macrophages and T lymphocytes, leading to fever, cytopenia, liver dysfunction, and potentially fatal multiorgan failure if untreated.

We report two cases of GS type 2 within the same family, revealed by MAS.

## Case presentation

Case 1

The patient was a 10-year-old girl from non-consanguineous parents, whose healthy mother had gray hair with a silver sheen since birth. She was admitted with a fever for one week, abdominal pain, and a rapid deterioration in her general condition. She did not have any psychomotor delay.

**Figure 1:**
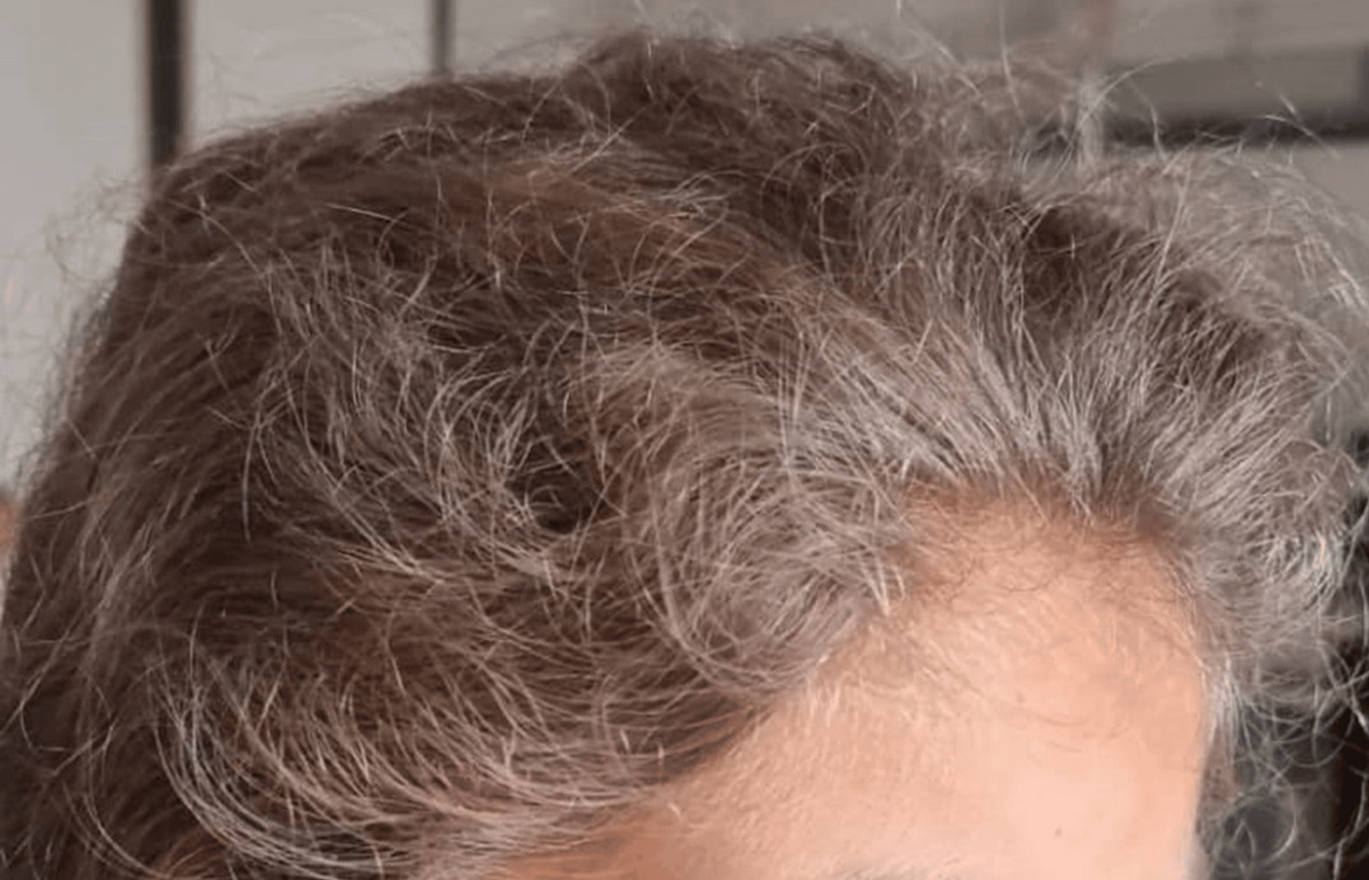
Picture of the first patient showing gray hair with silver highlights.

**Figure 2:**
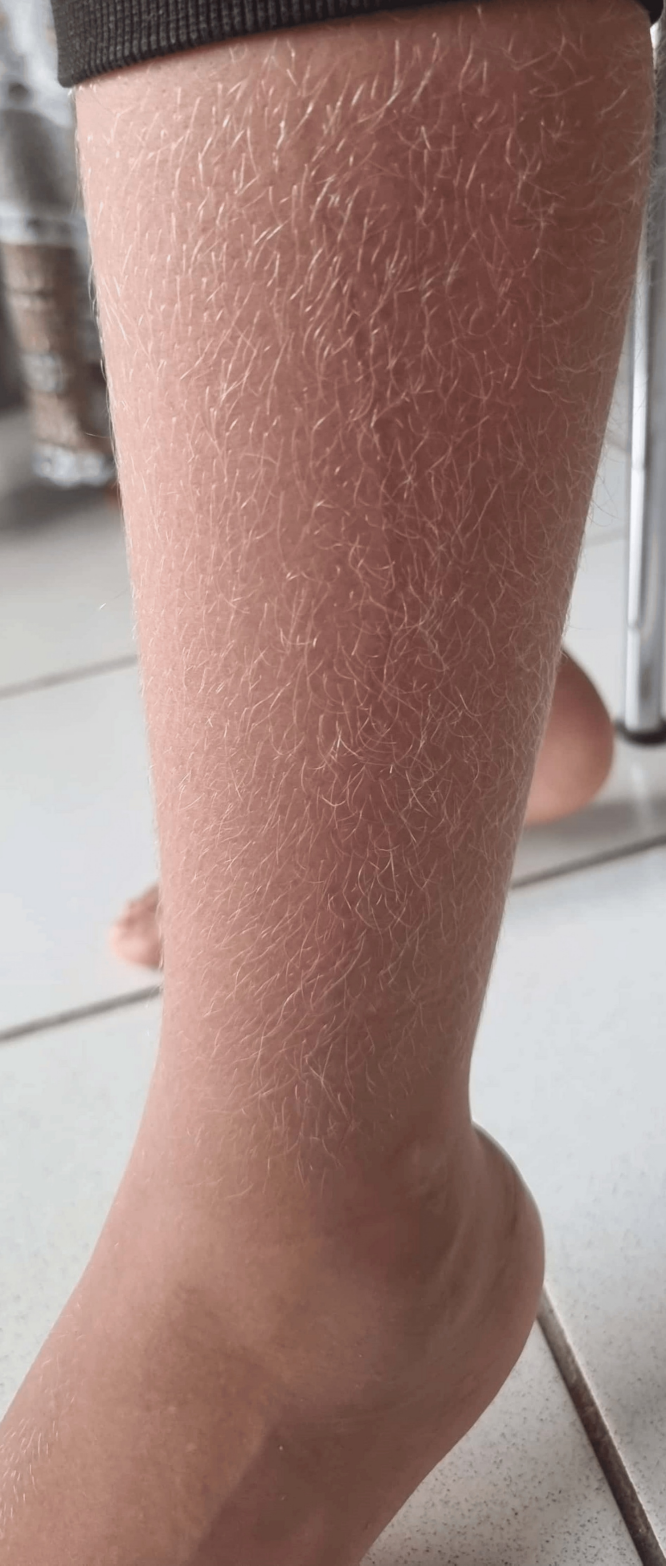
Picture of the first patient showing hair depigmentation.

On clinical examination, the patient was asthenic and febrile at 40°C, with hepatomegaly and splenomegaly, both 4 cm from the costal margin, associated with bilateral cervical adenopathies without inflammatory signs. On skin examination, there was a gray coloration of the hair with a silver sheen (Figure 1) and depigmentation of the hair (Figure 2) as well as petechiae on both lower limbs. The neurological examination was normal.

Biologically, pancytopenia was observed, with an aregenerative microcytic anemia. She had a hemoglobin of 5.8 g/dl, a mean corpuscular volume of 68 fl, and a reticulocyte count of 40 G/L, with leukopenia of 3.1 G/L, severe neutropenia of 0.32 G/L, and thrombocytopenia of 86 G/L. The blood smear showed a few schistocytes. The nucleated cells had a normal appearance and did not contain abnormal intracytoplasmic granules, eliminating Chediak-Higashi syndrome.

The fibrinogen level had collapsed to 1.34 G/L, the prothrombin level to 91%, and the activated partial thromboplastin time to 44 seconds for a control at 36 seconds. Alkaline phosphatase was at 521 U/L, and the gamma-glutamyl transferase level was at 98 U/L. Alanine aminotransferase (ALT) was at 384 IU/L or 10 times of normal, with aspartate aminotransferase (AST) at 233 IU/L or six times of normal. The ferritin level was high at 3092 µg/L, and the triglyceridemia was high at 3.6 mmol/L (Table 1). Renal function was normal. The Coombs test was negative. Cytobacteriological examination of the sputum revealed the presence of *Staphylococcus aureus* on the culture. Viral serologies were negative. Blood cultures and cytobacteriological examination of urine were sterile.

**Table 1:** Laboratory results of our two patients.

Lab test	Patient 1	Patient 2	Normal range
Hemoglobin	5.8 g/dL	10 g/dl	12–16 g/dL
Mean corpuscular volume	68 fL	70 fL	80–100 fL
Reticulocyte count	40 G/L	50 G/L	20-100 G/L
Leukocyte count (WBC)	3.1 G/L	4200 G/L	4.0–11.0 G/L
Neutrophils	0.32 G/L	1.4 G/L	1.8–8.0 G/L
Monocytes	–	8.8 G/L	0.2–1.0 G/L
Platelet count	86 G/L	43 G/L	150–400 G/L
Fibrinogen	1.34 G/L	1.7 G/L	2.0–4.0 G/L
Prothrombin (PT) level (PT activity)	91%	69%	70–100%
Activated partial thromboplastin (aPTT)	44 s (control: 36 s)	37 s (control: 36 s)	~25–35 s
Alanine aminotransferase (ALT)	384 IU/L	101 IU/L	7–56 IU/L
Aspartate aminotransferase (AST)	233 IU/L	113 IU/L	10–40 IU/L
Alkaline phosphatase (ALP)	521 U/L	–	44–147 U/L
Gamma-glutamyl transferase (GGT)	98 U/L	–	15–85 U/L
Ferritin	3092 µg/L	196 µg/L	20–200 µg/L
Triglycerides	3.6 mmol/L	2.65 mmol/L	<1.7 mmol/L
Lactate dehydrogenase (LDH)	–	617 IU/L	140–280 IU/L
C-reactive protein (CRP)	–	45 mg/L	<5 mg/L

**Figure 3:**
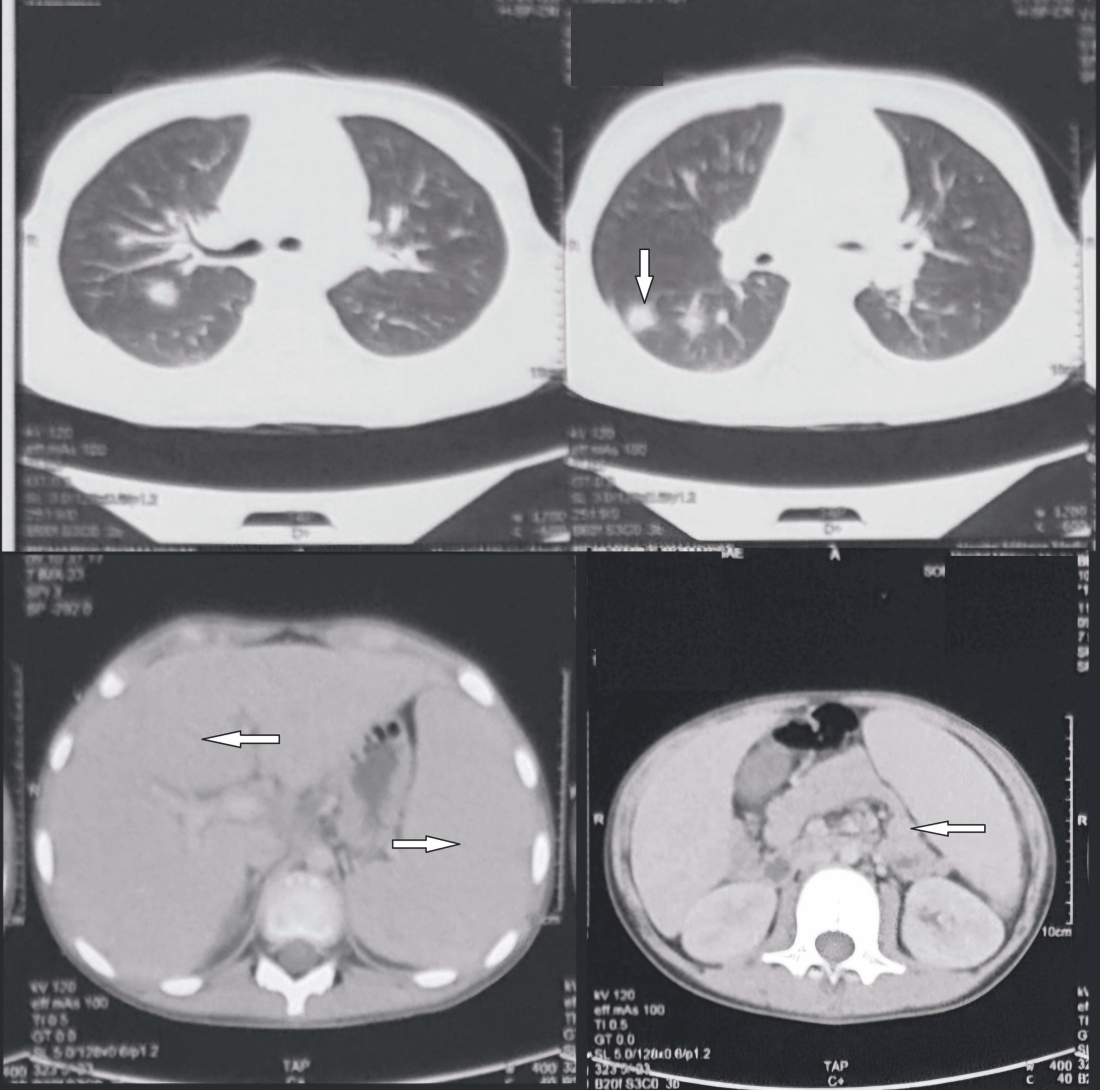
Images from the cervical and thoraco-abdomino-pelvic CT scan of the first patient showing homogeneous hepatosplenomegaly with supra and subdiaphragmatic adenopathies and bilateral mixed pulmonary nodules.

A cervico-thoraco-abdomino-pelvic CT scan revealed homogeneous hepatosplenomegaly, without focal lesions, with supra and subdiaphragmatic adenopathies as well as bilateral mixed pulmonary nodules (Figure 3).

**Figure 4:**
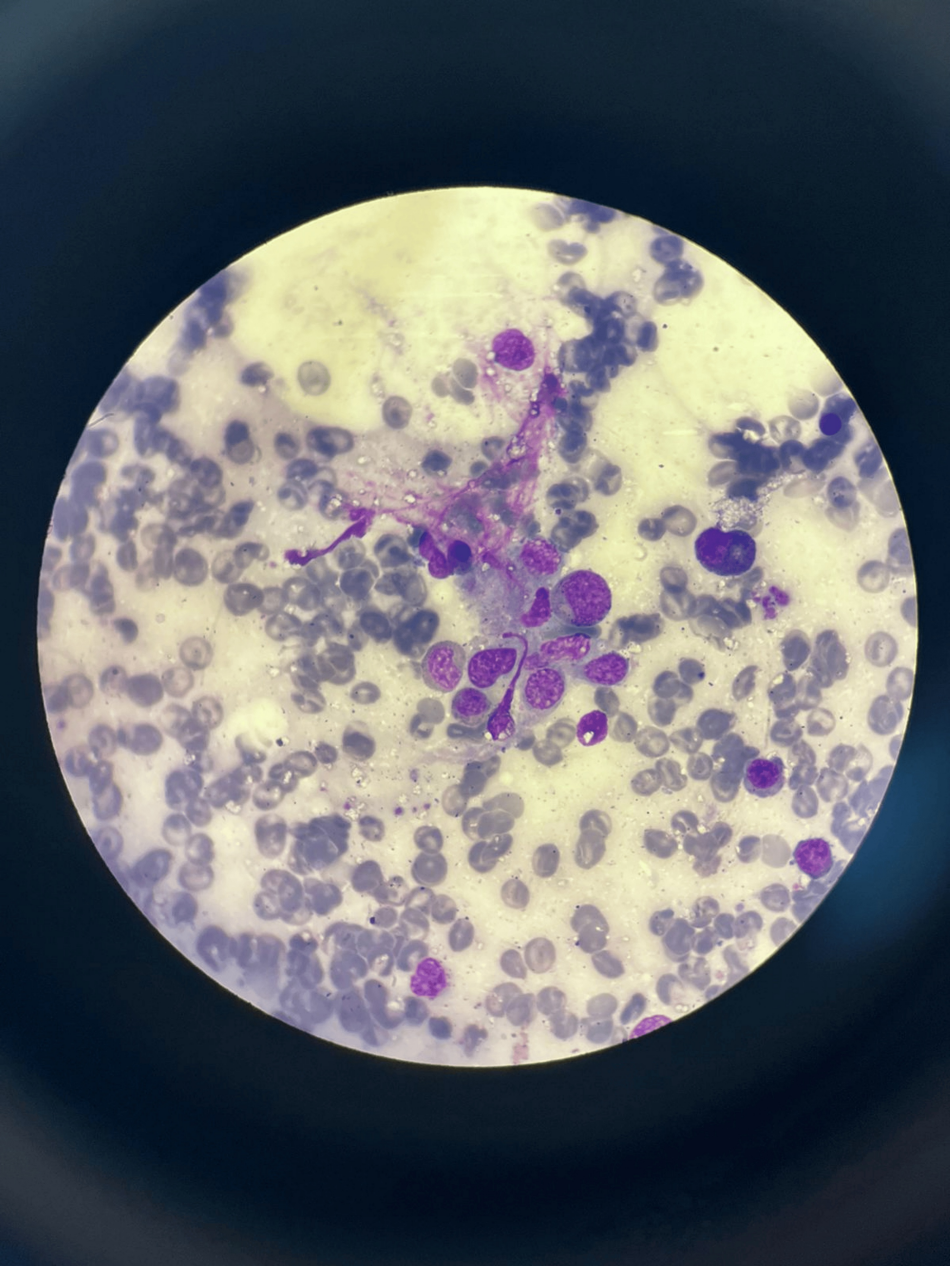
Image of hemophagocytosis in the myelogram performed on the first patient.

Given this picture of febrile pancytopenia associated with stigmata of MAS, a myelogram was performed, showing rich bone marrow and images of hemophagocytosis (Figure 4), with the absence of blastic or malignant cells.

**Figure 5:**
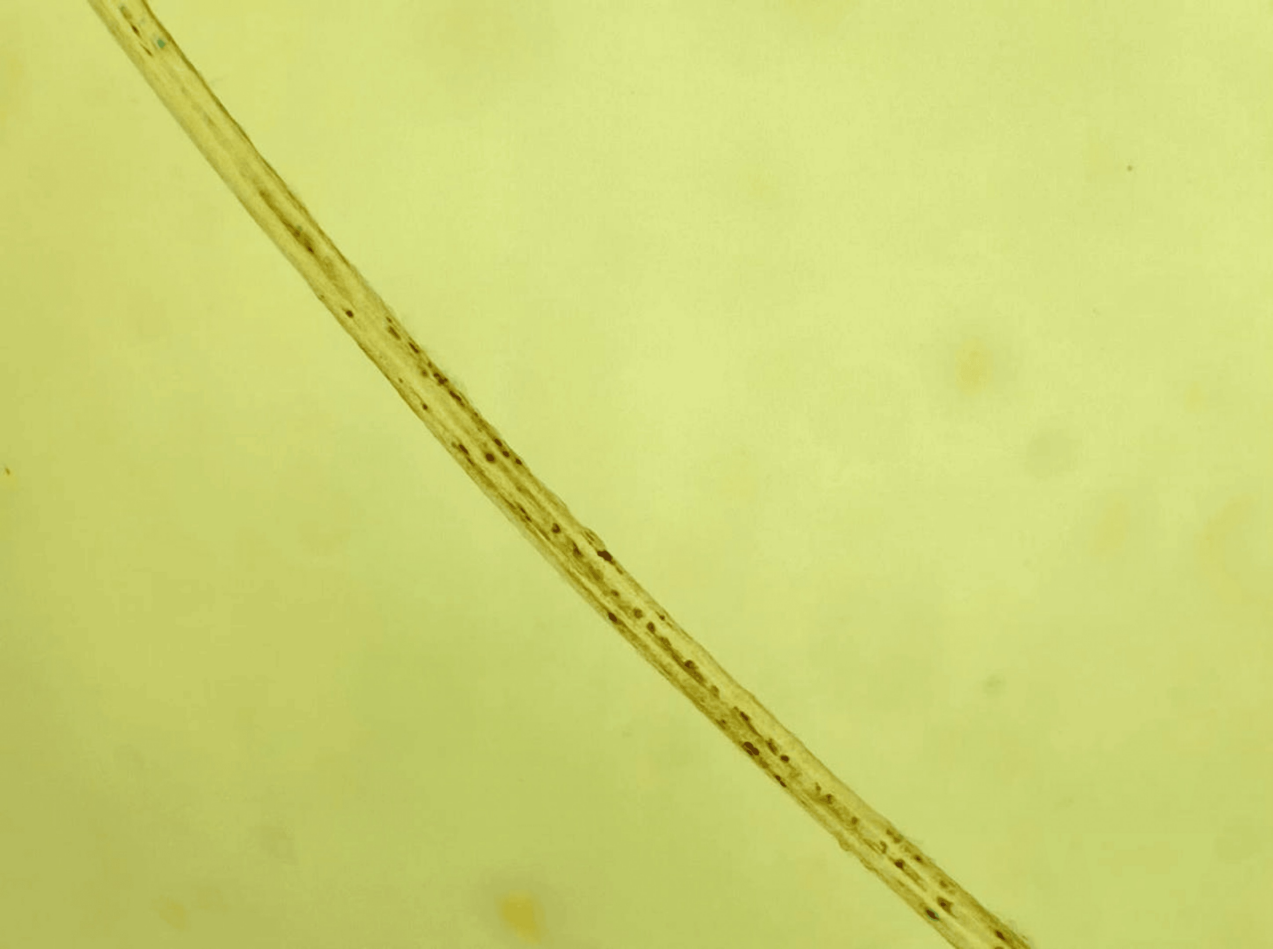
Optical microscope examination of a sample of the first patient's hair showing the presence of large, irregular clumps of pigment in its sheath.

Light microscopic examination of a hair sample revealed the presence of large, irregular lumps of pigment within its sheath (Figure 5).

Molecular biology showed a mutation in the RAB27A gene, confirming the diagnosis of GS type 2.

In front of the primary MAS, the patient was put on a corticosteroid bolus. The evolution was marked by a convulsive status epilepticus. She was hospitalized in intensive care, intubated and ventilated, and received broad-spectrum antibiotic therapy, immunoglobulins, and then corticosteroid therapy. The patient developed a pneumo-renal syndrome with alveolar hemorrhage and persistent renal failure. She received several hemodialysis sessions and was put on anti-CD20 for four weeks with corticosteroid boluses.

Human leukocyte antigen (HLA) typing was performed on the siblings for bone marrow transplantation. The only compatible family member was the brother, who had the same syndrome. The outcome was fatal, with the patient dying.

Case 2

A seven-year-old boy, the brother of our patient, was admitted for bilateral cervical swelling that had been developing for five days in a feverish context. He did not present any psychomotor delay.

**Figure 6:**
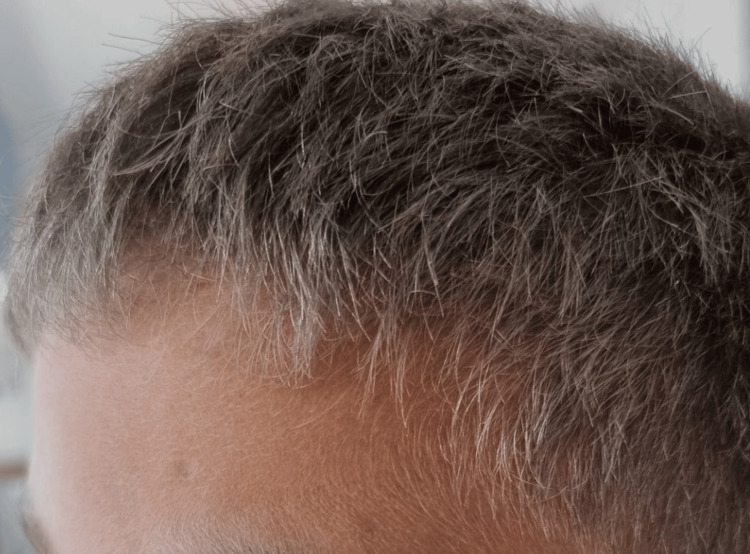
Picture of the second patient showing gray hair with silver highlights.

On clinical examination, the patient was asthenic and febrile at 40°C and tachycardic at 158 ​​bpm, without signs of peripheral hypoperfusion. He presented a large bilateral cervical swelling of 4 to 5 cm in diameter and firm consistency, associated with erythematous pultaceous angina. Abdominal examination revealed hepatomegaly at 12 cm and splenomegaly at 2 cm from the costal margin. On skin examination, gray hair color with silver highlights was found (Figure 6), with depigmentation of the hair. The neurological examination was normal.

Biologically, bicytopenia was observed, with neutropenia at 1.4 G/L, monocytosis at 8.8 G/L, and thrombocytopenia at 43 G/L. The nucleated cells did not have abnormal intracytoplasmic granules, eliminating Chediak-Higashi syndrome.

The fibrinogen level was decreased to 1.7 g/L, the prothrombin level to 69%, and the activated partial thromboplastin time to 37 seconds for a control at 36 seconds. ALT was at 101 IU/L or three times of normal, and the AST was at 113 IU/L or four times of normal. Renal function was normal. Serum ferritin was at 196 µg/L, and triglyceridemia was elevated to 2.65 mmol/L and lactate dehydrogenase (LDH) to 617 IU/L. C-reactive protein was at 45 mg/L (Table 1). Epstein-Barr virus (EBV) serology showed positive anti-VCA IgM, suggesting a primary EBV infection. Blood cultures and cytobacteriological examination of urine were sterile.

**Figure 7:**
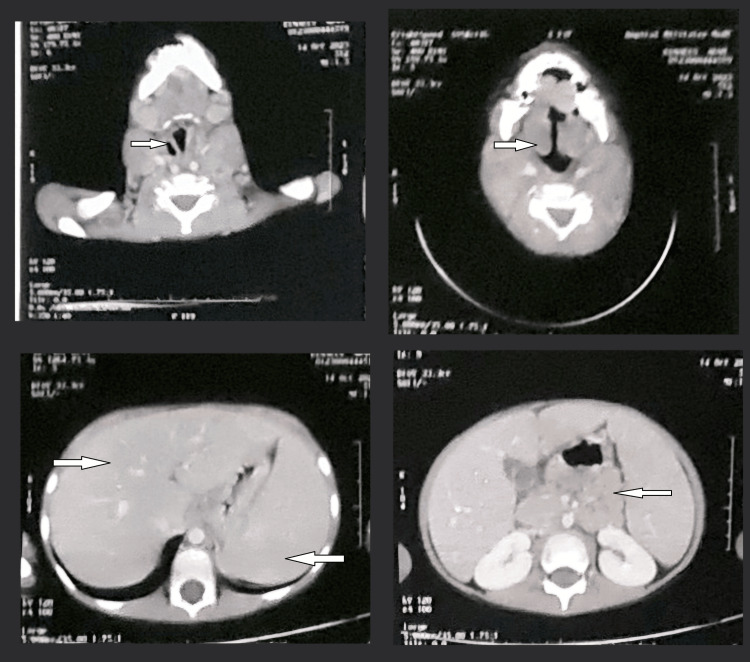
Images from the cervical and thoraco-abdomino-pelvic CT scan of the second patient showing thickening of the nasopharynx, reducing the airway with bilateral cervical adenopathies and homogeneous hepatosplenomegaly with supra and subdiaphragmatic adenopathies.

Cervical CT scan showed complete thickening of the nasopharynx, reducing the airway with bilateral cervical adenopathies (Figure 7). A thoraco-abdomino-pelvic CT scan showed homogeneous hepatosplenomegaly with supra and subdiaphragmatic adenopathies (Figure 7).

Given this picture of febrile bicytopenia associated with stigmata of MAS, a myelogram was performed, showing rich bone marrow and images of hemophagocytosis, with the absence of blastic or malignant cells.

Examination of a hair sample under a light microscope revealed the presence of large, irregular lumps of pigment within its sheath.

The search for the RAB27A gene mutation could not be carried out because the progression was rapidly fatal, with the patient dying after seven days of hospitalization.

## Discussion

GS is a fatal autosomal recessive disorder, first described by Griscelli et al. in 1978 [1]. It is classified into three types based on genetic and molecular characteristics. Mutations in the MYO5A, RAB27, and MLPH genes are responsible for the distinct manifestations of types 1, 2, and 3, respectively.

It is characterized by partial albinism and a silvery sheen to the hair, found in our two patients. This aspect is common to all types of GS. In type 1 GS, it also manifests itself by various neurological disorders, dominated by severe and early psychomotor delay. In type 3 GS, it is isolated; this was observed in the mother of our two patients. The diagnosis is often late, overall survival is similar to the general population, and no treatment is necessary [2].

Type 2 GS is caused by a mutation in the Rab27a (RAB27A) gene located on chromosome 15q21. This mutation was found in our first patient. RAB27A is a guanosine triphosphatase that plays an essential role in the peripheral transport of melanosomes to neighboring cells, such as keratinocytes, and the exocytosis of granules in cytotoxic T lymphocytes. A mutation in the RAB27A gene results in partial albinism of the skin and hair, and uncontrolled T lymphocytes and macrophage hyperactivation, leading to macrophage activation syndrome.

Beyond its role in melanosome transport, RAB27A is essential for the docking and exocytosis of lytic granules in cytotoxic T cells and natural killer (NK) cells. Defects in this pathway lead to impaired killing of infected cells and sustained immune stimulation, explaining the uncontrolled cytokine release seen in macrophage activation syndrome. Experimental work also suggests that altered RAB27A function disrupts vesicle trafficking in neurons and glial cells, which may contribute to the neurological manifestations sometimes observed in GS type 2.

Type 2 GS is, in fact, characterized by an immune deficiency responsible for repeated infections. It constantly evolves toward a so-called "acceleration" phase of formidable prognosis, which manifests itself by a rapidly fatal MAS [3]. Patients with GS type 2 may present neurological symptoms secondary to infiltration of the central nervous system (CNS) by activated hematopoietic cells. CNS involvement may be apparent from the initial presentation or occur at any time during the MAS, with symptoms including epileptic seizures, ataxia, facial paralysis, spasticity, irritability, gait disturbances, and coma. The occurrence of convulsive status epilepticus in our first patient may have been due to CNS infiltration. In patients without a specific family history, neurological deficits as the first clinical signs may delay accurate diagnosis, as symptoms are similar to other neurological diseases, such as primary (e.g., autoimmune encephalitis and CNS vasculitis) and secondary (e.g., infections and tumors) etiologies [4].

Although GS type 2 usually manifests in infancy or early childhood, some patients present later, occasionally with isolated neurological signs or a chronic relapsing course. Infections, particularly Epstein-Barr virus, cytomegalovirus, or bacterial sepsis, commonly act as precipitants of MAS. Awareness of these triggers is important for early recognition and prompt initiation of treatment.

In both our cases, the diagnosis of type 2 GS was made based on a set of clinical and biological arguments: oculocutaneous hypopigmentation, silvery reflection of the hair, absence of psychomotor delay, occurrence of MAS following an infection, and especially the pathognomonic appearance of the hair on microscopic examination.

When evaluating a child with silvery hair, oculocutaneous hypopigmentation, fever, and cytopenia, a systematic approach is required. Microscopic examination of the hair shaft remains a simple, rapid, and inexpensive tool for distinguishing GS from other hypopigmentation syndromes. Flow cytometry assessing NK-cell degranulation, along with genetic analysis, helps confirm the diagnosis and separate GS type 2 from disorders such as Chediak-Higashi syndrome, Hermansky-Pudlak type 2, and familial forms of hemophagocytic lymphohistiocytosis.

GS and Chediak-Higashi syndrome are the first diagnoses to be considered in a child with silver hair and partial albinism [5]. In our patients, the absence of giant granulations in the nucleated cells made it possible to eliminate Chediak-Higashi syndrome.

Regarding prognosis and treatment methods, chemotherapy (etoposide) and, more recently, antilymphocyte serum and cyclosporine have allowed short-term remission to be achieved. Intrathecal injections of methotrexate are effective in cerebral invasions [6,4].

Recently, ruxolitinib (RUX), an oral selective JAK1/2 inhibitor, has shown great promise in mouse models of primary and secondary MAS, including improvements in central nervous system involvement [7-9]. The use of RUX as a bridge to allogeneic hematopoietic stem cell transplantation (allo-HSCT) for refractory primary MAS has also been reported [9]. Furthermore, several clinical trials evaluating RUX in the treatment of MAS are ongoing, and preliminary data suggest that RUX is effective and safe in these settings [4].

Currently, MAS-directed therapy followed by hematopoietic stem cell transplantation (HSCT) is the only curative treatment for patients with primary MAS, including GS type 2. According to a large retrospective study of HSCT in children with GS type 2, neurological involvement before HSCT was an adverse predictor of survival, with an overall five-year survival rate of 50 ± 12.5% [4].

In our setting, however, essential targeted therapies, such as etoposide and ruxolitinib, were not available at the time of management. One of our patients additionally received anti-CD20 therapy (rituximab), but apart from that, treatment was limited to corticosteroids and supportive measures.

Allogeneic bone marrow transplantation could not be performed in our two patients, partly because of the absence of a compatible healthy donor and partly because the disease progressed rapidly to death.

Families affected by GS should receive detailed counseling on inheritance patterns and recurrence risk. Prenatal diagnosis through chorionic villus sampling or amniocentesis enables early detection in subsequent pregnancies, while preimplantation genetic testing may be discussed where available [10].

In our report, genetic counseling had not been offered to the parents prior to conception; however, after the diagnosis of GS was established in their children, we provided counseling, highlighting the autosomal-recessive inheritance, the 25% recurrence risk, and the options for prenatal or preimplantation diagnosis.

## Conclusions

GS is a rare disorder that is often challenging to diagnose due to its clinical similarities to malignancies, infectious diseases, and immunodeficiencies. Given its poor prognosis, GS should be considered in pediatric patients presenting with pancytopenia, splenomegaly, and hypopigmented hair, particularly in children from high-risk families, including those with consanguinity or a positive family history. Therefore, microscopic examination of hair and skin, along with genetic testing, is strongly recommended. Early diagnosis and treatment can significantly improve the chances of survival in affected individuals.

## References

[1] Perugu RK, Karra N, Shaik SS, Venigalla WC, G P, Maram MR (2023). Griscelli syndrome with hemophagocytic lymphohistiocytosis: a rare case report. Cureus.

[2] Manglani M, Adhvaryu K, Seth B (2004). Griscelli syndrome - a case report. Indian Pediatr.

[3] Pachlopnik Schmid J, Moshous D, Boddaert N (2009). Hematopoietic stem cell transplantation in Griscelli syndrome type 2: a single-center report on 10 patients. Blood.

[4] Zhang Q, Zhao YZ, Ma HH, Wang D, Zhang N, Li ZG, Zhang R (2021). Successful rescue of a lethal Griscelli syndrome type 2 presenting with neurological involvement and hemophagocytic lymphohistiocytosis: a case report. BMC Pediatr.

[5] Klein C, Philippe N, Le Deist F (1994). Partial albinism with immunodeficiency (Griscelli syndrome). J Pediatr.

[6] Aricò M, Zecca M, Santoro N (2002). Successful treatment of Griscelli syndrome with unrelated donor allogeneic hematopoietic stem cell transplantation. Bone Marrow Transplant.

[7] Das R, Guan P, Sprague L (2016). Janus kinase inhibition lessens inflammation and ameliorates disease in murine models of hemophagocytic lymphohistiocytosis. Blood.

[8] Albeituni S, Verbist KC, Tedrick PE, Tillman H, Picarsic J, Bassett R, Nichols KE (2019). Mechanisms of action of ruxolitinib in murine models of hemophagocytic lymphohistiocytosis. Blood.

[9] Zhao Y, Shi J, Li X, Wang J, Sun J, Zhou J, Huang H (2020). Salvage therapy with dose-escalating ruxolitinib as a bridge to allogeneic stem cell transplantation for refractory hemophagocytic lymphohistiocytosis. Bone Marrow Transplant.

[10] Durandy A, Breton-Gorius J, Guy-Grand D, Dumez C, Griscelli C (1993). Prenatal diagnosis of syndromes associating albinism and immune deficiencies (Chediak-Higashi syndrome and variant). Prenat Diagn.

